# Chinese international students’ conceptualizations of wellbeing: A prototype analysis

**DOI:** 10.3389/fpsyg.2022.939576

**Published:** 2022-08-24

**Authors:** Lanxi Huang, Margaret L. Kern, Lindsay G. Oades

**Affiliations:** Centre for Wellbeing Science, Melbourne Graduate School of Education, The University of Melbourne, Parkville, VIC, Australia

**Keywords:** prototype analysis, wellbeing, Chinese international students, lay conceptualizations, mental health, tertiary education

## Abstract

Wellbeing can mean different things to different people, even in the same culture with the same language. People living at the intersection of two languages and cultures, such as Chinese students studying in an English-speaking nation, not only speak a different language than their host country, but also may have different conceptualizations of wellbeing itself. This study investigated Chinese international students’ (aged 18–39, *N* = 123) conceptualizations of wellbeing using a modified prototype analysis, which provided insights on people’s underlying structure of the construct as revealed through language. Chinese international students’ conceptualizations of wellbeing were prototypically structured; key components of wellbeing included positive relationships, security, positivity/optimism, physical health, and self-strength. The findings broaden the understanding of layperson wellbeing conceptualizations, provide insights into the wellbeing related concepts and language that are most used by international Chinese students, and inform strategies that tertiary education institutions might adopt to effectively support Chinese international students’ wellbeing.

## Introduction

Within academia, numerous ways of defining, measuring, and evaluating wellbeing have been developed (OECD, 2013). However, compared to academic conceptualizations of wellbeing, laypeople often hold different understandings of this construct (e.g., McMahan et al., 2013; Hone et al., 2015), which are influenced by micro individual factors and macro cultural contexts (Furnham and Cheng, 2000; Bowling and Gabriel, 2007; Brady, 2009). International students sit at the nexus of these micro and macro factors, as micro aspects arising from their home culture intersect with and at times clash with macro aspects of the host culture. Young adulthood at the tertiary education level is a critical period of transition that often brings dramatic changes, including risk of mental illness (Cvetkovski et al., 2012; McAuliffe et al., 2012; Said et al., 2013; Furber et al., 2015), with increased risk for international students (Forbes-Mewett and Sawyer, 2016). Thus, it is critical to better understand how international students conceptualize wellbeing. Addressing this need, the current study investigates the understanding and narrative of wellbeing by Chinese international students living in Australia, utilizing a mixed-method approach.

### Lay concepts of wellbeing

In this study, we focus specifically on conceptualizations of subjective wellbeing. From the subjective perspective, most of the current wellbeing models draw upon one or more of four philosophical traditions (Utilitarian, Virtue, Hedonic, and Eudaimonic), integrate hedonic and eudaimonic dimensions (e.g., Kashdan et al., 2008; Waterman et al., 2010; Delle Fave et al., 2011), incorporate virtues and strengths as core contributors to wellbeing, and aim to create the greatest happiness for the largest number (Keyes et al., 2002; Seligman, 2012).

Despite academic debates over wellbeing as a construct, only a limited number of studies have considered how laypeople understand and define wellbeing. Lay theory assumes that people establish a theory-consistent patterns of interactions, which impacts cognitive process phenomena, affects emotional arousal, and guides behaviors in line with their conceptualizations (Hong et al., 2001; Murphy and Dweck, 2010). Lay conceptualizations of wellbeing are a type of lay theory, which suggests that lay people develop their own intuitive theories to understand, interpret, experience, and behave related to their wellbeing within the environment (McMahan et al., 2013). Compared to academic notions of wellbeing, lay conceptualizations of wellbeing often differ from theoretical models, as they incorporate cultural beliefs, personal values, and diverse experiences (Brady, 2009; Wong et al., 2011; Joshanloo, 2019; Karnaze, 2019).

A growing number of studies have directly examined lay wellbeing conceptualizations for specific populations. Studies find that conceptualizations are influenced by age (Fattore et al., 2007; Huebner et al., 2012), cultural background (Jugureanu and Hughes, 2010; Delle Fave et al., 2016; Jen, 2017), and occupation (Blair et al., 2008; Fortune and Kennedy-Jones, 2014). For example, Sastre (1999) found that physical health, family relationships, and self-acceptance resonated with adults across age groups. Another study found that New Zealand adolescents valued enjoyment, feeling safe, being kind/helpful, and having a sense of satisfaction (Bharara et al., 2019). For teachers and lawyers, physical health, work-life balance, and feeling valued were central characteristics (Hone et al., 2015), whereas mental, emotional and general health, and personal relationships were vital for critical care nurses’ sense of wellbeing (Jarden et al., 2019).

Although the components identified in these studies align to some extent with existing theoretical wellbeing models, the theoretical models miss nuances that resonate with different populations. Misalignment between academic and lay conceptualizations of wellbeing can negatively impact intervention efforts, as interventions might miss the needs of the population of interest. Thus, further consideration of wellbeing conceptualizations for specific populations is needed.

### Chinese international students as a unique population

Here, we focus specifically on Chinese international students living in Australia. International students are critical to the economic, intellectual, and social culture of the host country (e.g., Ziguras and Law, 2006; Luo and Jamieson-Drake, 2013; Sawir, 2013). In Australia, international education is the fourth largest export industry; in 2019, Australia attracted more than 950,000 international students from 204 countries (Australian Government, 2020). Among all international students, over a quarter of students come from China (Green and Ferguson, 2015). Across countries, students in many university disciplines experience elevated levels of psychological distress due to academic, financial, and interpersonal factors (Rickwood et al., 2016; Matthewman et al., 2018). Compared to domestic students, international students face more numerous/severe challenges and adjustment problems as they try to navigate the foreign environment (Nilsson et al., 2004), especially when there are significant differences between the home and host culture, language, social structures, quality of life, and sense of safety, as is the case for Chinese students in Australia. Further, despite all of the existing services that Australian universities provide, supports and activities on campus generally are based on Western experiences and perspectives, which may not align with Chinese international students’ conceptualizations of wellbeing, such that supporting resources misalign with the needs that students have. In this study, we focus on wellbeing and lay conceptualization of wellbeing instead of mental illness or mental health. Therefore, mental health and wellbeing are not used interchangeable or synonymously here.

Student wellbeing is positively related to their capacity for academic achievement, ability to thrive in the tertiary environment, and later attitudinal and career outcomes (Field et al., 2015; Nelson et al., 2015). The question of how tertiary educational institutions support Chinese international students’ wellbeing becomes crucial for not only meeting students’ wellbeing needs, but also to build a good reputation in the competitive international education market. To support international students well, it is important to first understand students’ conceptualizations of wellbeing. An initial study found that Chinese international students perceived that physical health, mental health, security, relationship support, and prosperity are the most important constitutes of wellbeing, which differ to some extent from the current academic-accepted models of wellbeing (Huang et al., 2020). Extending this research, the current study uses a prototype analysis approach to further elucidate Chinese international student lay conceptualizations of wellbeing.

### The prototype analysis approach

Rosch (1975) introduced the “prototypical approach” as a method for conceptual inquiry, which shed light on differentiating the natural language of a concept from the classical view of the concept. In this approach, the *classical view of concept structure* is that all the category memberships or components within a concept are sufficient and necessary, thus sharing equal levels of importance (Rosch, 1975). For instance, if wellbeing is defined in terms of emotions, engagement in life, positive relationships with others, having a sense of meaning, and feelings of accomplishment (Seligman, 2012), wellbeing occurs when these five dimensions are fulfilled, regardless of a person’s values or background. In contrast, Rosch (1975) argued that the *natural language constructed concept* is cognitively processed by a prototype with central features, with decreased importance for peripheral features. In other words, categories or components have a ranked order of importance, such that some are more critical and central to the core concept than others. For instance, a person might define wellbeing more in terms of positive emotion, high-quality relationships and accomplishment, whereas engagement and meaning in life would be less relevant to their definition.

In Rosch’s approach, two conditions must be met to demonstrate the prototype structure of a concept. Firstly, people must be able to list and rate the centrality of components to a given concept. That is, people can identify some components that are more central to their definition of the concept than other components are. Secondly, the differential centrality of components affects people’s cognitive processing of the relevant concept, as revealed by language (Rosch, 1975). That is, people’s understanding depends upon the ordering of different components, which can further provide insights on people’s perspectives, cognitive and emotional responses, and related behaviors. Several methods have been developed to verify the second condition, such as recall memory (Kearns and Fincham, 2004) and reaction time (Gregg et al., 2008). The greater the difference between the convergence of measures in internal structure represents the higher possibility that the concept is prototypically structured.

Lambert et al. (2009) suggested that prototype analysis is particularly suitable for blurry components within natural language. The analysis can also be used to compare different conceptualizations across cross-cultural contexts to identify differences that occur across different populations (Morgan et al., 2014). Prototype analysis has previously been used to explore laypeople’s conceptualizations of wellbeing (Hone et al., 2015) as well as other psychological constructs such as forgiveness (Kearns and Fincham, 2004) and gratitude (Lambert et al., 2009). Hone et al. (2015)’s studies suggested that wellbeing is prototypically organized and New Zealand worker’s perspective of wellbeing is different from researchers’ models. The approach also helped identify activities that people engage in to maintain and promote wellbeing. However, it is unclear whether the same structure occurs for Chinese international students.

Notably, prototype analysis typically has been used to identify the common conceptualizations that occur across a population. The analysis often involves a series of studies with different samples from the defined population. While this has the advantage of identifying central aspects across that population, the nomothetic approach ignores individual variation. Wellbeing is a value-based concept (Alexandrova, 2017; Kern et al., 2020), arising in part from the unique way that a person adjusts to their environment (Allport, 1937). From an idiographic perspective, even as common conceptualizations of wellbeing might arise across a specific population, the centrality structure may be unique to the individual. Thus, beyond identifying nomothetic conceptualizations of wellbeing, alternative insights might arise from identifying idiographic variations within that population.

### The present study

Wellbeing science faces the crucial question of how wellbeing should be defined (Kern et al., 2020). While some studies have examined lay conceptualizations of wellbeing, little is known about the conceptualizations of Chinese international students, which is critical for proactively supporting wellbeing and providing adequate services that meet the needs that students have. The current study uses a modified prototype analysis to examine the wellbeing conceptualizations of Chinese international students living in Australia. Prototype analysis provides a method to move away from imposed Western conceptualizations to reveal how the students themselves conceptualize wellbeing, the extent to which is it prototypically organized, and alignment with existing models that currently inform wellbeing care the extent to which Chinese students’ perspectives align with academic models of wellbeing. Addressing nomothetic concepts, analyses identified common concepts that arose across participants, across three steps. Addressing potential idiographic variation, in this modified version, the same participants completed the three steps of the study, identifying central components of wellbeing specific to the participants themselves, rather than using different participants for each of the three steps.

## Materials and methods

### Procedure overview

Potential participants were recruited through Chinese International Students Associations and other similar associations/student groups from the main eight universities in Melbourne, Australia. The associations and groups were asked to display a flyer created for the recruitment of participants and to provide a link to the study in their WeChat groups and Facebook groups. Interested students followed a link that provided details on the study. Consenting individuals were immediately taken to the survey. Participants could choose to complete the survey in either the English or Chinese (Mandarin) version of the survey, and for questions, they could respond in English, Chinese, or a mix of English and Chinese. Data were collected between September 2019 and February 2020.

The survey first asked participants basic demographic information, including gender, age, education level and status, length of stay, and English proficiency level (reading, writing, comprehension). English test scores were converted into International English Language Testing System (IELTS) scores based on the equivalent standard set by the university’s admission requirements and official score conversions (see Supplementary Material 1).

The study intended to focus on a normal (non-clinical) population. To screen for potential psychological distress, participants were required to respond to the six-item Kessler Distress Scale (Kessler et al., 2003). While the scale is not diagnostic of mental illness, the scale has been used as a screening tool in several population-based studies in Australia, with scores 19 and above indicating probable severe mental distress. The Qualtrics survey software automatically calculated their score, and if a potential participant scored at 19 and above, they were directed to a page that thanked them for their interest, noted that they were ineligible for the study, and provided a series of resources for further professional support and service. Individuals with scores below 19 were directed on to the rest of the survey, which took them through the three steps, described in detail below.

This research was conducted in accordance with the University of Melbourne’s ethics review policies (protocol #1954456.1). The study utilized an anonymous survey and participants could withdraw at any time while completing the survey. At the end of the survey, a series of recourses were provided for references, such as a Mandarin/English speaking psychologist, the university’s counseling service, and external professional support like Lifeline Australia.^1^

### Participants

To be included in the study, participants had to be a Chinese international student who was (1) studying at a tertiary education institution in Melbourne, Australia; (2) at least 18 years old; and (3) had lived in Melbourne for a minimum of 3 months and a maximum of 4 years. The living requirement aimed to ensure that participants were beyond the immediate transition period of living abroad and had significant experiences across both Australian and Chinese cultures.

Following other wellbeing prototype analytic studies (e.g., Hone et al., 2015; Bharara et al., 2019), we aimed to include 100 participants across the three steps. Of 300 individuals who showed interest in being a part of the study, 228 responded to the online survey. Forty-seven were excluded due to not meeting the inclusion criteria, and 32 were excluded due to showing signs of potential psychological distress. Of those who met the inclusion criteria, 123 students completed Step 1, 117 students completed Step 2, and 90 students completed Step 3. As summarized in Table 1, the sample was about one-third male and two-third female. Participants were primarily between 21 and 29 years old. For English proficiency level, approximately 60% of students were at the good and competent levels regarding the comprehensive score, at an excellent or good level regarding the reading score, and a competent or modest level regarding the writing score. About three-fourths of students had lived in Melbourne for at least 1 year.

**TABLE 1:** Participant demographic characteristic.

Characteristic	Category		Step 1 *n* (%)	Step 2 *n* (%)	Step 3 *n* (%)
Gender	Male		35 (28.5%)	33 (28.2%)	29 (32.2%)
	Female		88 (71.5%)	84 (71.8%)	61 (67.8%)
Age	18–20		30 (24.4%)	28 (23.9%)	17 (18.9%)
	21–29		84 (68.3%)	80 (68.4%)	65 (72.2%)
	30–39		9 (7.3%)	9 (7.7%)	8 (8.9%)
Education level	High school graduate, diploma/equivalent		30 (24.4%)	28 (23.9%)	23 (25.6%)
	Undergraduate degree		33 (26.8%)	33 (28.2%)	22 (24.4%)
	Graduate degree		59 (48.0%)	55 (47.0%)	45 (50.0%)
Education status	Foundation course/university credit		18 (14.6%)	17 (14.5%)	12 (13.3%)
	Bachelor’s degree		37 (30.1%)	36 (30.8%)	24 (26.7%)
	Graduate certificate or diploma		6 (4.9%)	6 (5.1%)	5 (5.6%)
	Master’s degree		36 (29.3%)	35 (29.9%)	28 (31.1%)
	Doctorate degree		25 (20.3%)	23 (19.7%)	21 (23.3%)
English level	Comprehensive	Excellent	14 (11.4%)	14 (12.0%)	10 (11.1%)
		Good	40 (32.5%)	37 (31.6%)	28 (31.1%)
		Competent	41 (33.3%)	39 (33.3%)	32 (35.6%)
		Modest	8 (6.5%)	8 (6.8%)	8 (8.9%)
	Reading	Excellent	31 (25.2%)	31 (26.5%)	23 (25.6%)
		Good	37 (30.1%)	34 (29.1%)	24 (26.7%)
		Competent	24 (19.5%)	22 (18.8%)	21 (23.3%)
		Modest	7 (5.7%)	7 (6.0%)	6 (6.7%)
	Writing	Excellent	8 (6.5%)	8 (6.8%)	7 (7.8%)
		Good	20 (16.3%)	19 (16.2%)	13 (14.4%)
		Competent	51 (41.5%)	47 (40.2%)	37 (41.1%)
		Modest	20 (16.3%)	20 (17.1%)	17 (18.9%)
Length of stay	3 months – 12 months		21 (17.1%)	17 (14.5%)	19 (21.1%)
	13 months – 24 months		39 (31.7%)	39 (33.3%)	24 (26.7%)
	25 months – 36 months		32 (26.0%)	30 (25.6%)	20 (22.2%)
	37 months – 48 months		28 (22.8%)	28 (23.9%)	24 (26.7%)

### Procedure

The study involved three steps. In Step 1, participants listed the components regarding wellbeing in a free-response format. In Step 2, participants rated the centrality (or importance) of the components mentioned in step 1. Step 3 explored differences in descriptions of high and low levels of wellbeing.

#### Step 1: Free listing of prototypic wellbeing components

Step 1 aimed to compile a list of wellbeing components. Participants were asked to list as many components and indicators of wellbeing as they could in an open-question format. Following the demographic information questions, participants were given the following instructions (adapted from Hone et al., 2015; Bharara et al., 2019), with no time limit for their response:

*This is a study on what Chinese international students think of when they consider the word wellbeing. For example, if you were asked to list the components and indicators of fear, you might write: possible danger occurs, attention is focused on the threat, the heart beats wildly, the person runs as fast as they can. In the current study, we are not interested in fear but in the characteristics of wellbeing. Imagine that you are explaining this term to someone who has no experience of wellbeing and answer the following question: What, in your opinion, are the key components and indicators of wellbeing? Please list as many as you can. You can write in English, Chinese, or the mix of both languages.*

#### Step 2: Ranking the components

Step 2 aimed to explore whether conceptualizations of wellbeing possess a prototypical feature, with a central and peripheral structure among the components. The same group of participants were next shown the components that they had listed in Step 1, and were asked to rank how important or unimportant they think each one is. Specifically, participants were given the following instructions (adapted from Hone et al., 2015; Bharara et al., 2019), with no time limit for their response:

*Now, think about each of these components that you listed. Drag and drop these components, indicating which one is most important (on the top) down to which is least important (on the bottom). We would like you to think not only about your own experiences with wellbeing, but the concept of wellbeing in general – what you think are its defining components. Don’t worry about why you think something is or isn’t central.*

#### Step 3: Descriptions of high and low levels of wellbeing

Step 3 aimed to identify whether there are differences between how participants conceptualize high and low levels of wellbeing, identifying the extent to which the people’s perceptions of wellbeing would be affected by the centrality of components. Following Step 2, participants were asked to write two narratives, first describing a person with a high level of wellbeing, and then describing a person with a low level of wellbeing. Participants were given the following instructions, with no time limit for their response:

*In this question, we would like to ask you to write two paragraphs, describing a person whom you think has a high wellbeing level and a person with low wellbeing level. You can write in English, Chinese, or the mix of both languages.*

### Analytic approach

#### Step 1

Step 1 identified the wellbeing components mentioned by participants. As participants used English, Chinese, or a mix of languages, all Chinese words and phrases were first translated into English. The translation process was conducted with the assistance of a professional translator certified by the National Accreditation Authority for Translators and Interpreters (NAATI) Australia to minimize meaning distortion and subjective bias. Analyses were then conducted on the English data, following Fehr’s (1988) procedure for analyzing the free-listing responses. The first author identified the initial set of linguistic units, grouped the units into categories, and grouped the categories into representative components. The second and third author reviewed all codes and resolved any uncertainty or discrepancies, and further finalized the component list.

More specifically, monolexemic linguistic units which were easily recognizable were first identified and extracted. For instance, responses such as “peace,” “health,” and “autonomy” were coded as distinct linguistic units. Words that were preceded by modifiers or attributive words were coded by the representative word. For instance, “exercise regularly” was coded as “exercise,” “keep calm” was coded as “calm,” and “wild interests” was coded as “interests.”

Phrases, identified based on sets of words that have a clear meaning, were coded as single represent linguistic units. For instance, the responses “work is secure” and “small things to enjoy in life, e.g., a nice dinner” were judged as phrases that convey single thoughts (rather than as separate words or capturing multiple thoughts) and were coded as the individual linguistic units “job security” and “enjoyment,” respectively. In contrast, we judged the response “eat and sleep well” as conveying two thoughts (eating well and sleeping well), which thus was divided into the distinctive linguistic units “eat” and “sleep.” Another example is “company with family and friends” were divided into “company with family” and “company with friends.” To maintain the richness of responses to the greatest extend possible, we were conservative in our codes (i.e., generating a greater number of more specific codes, rather than a small number of broad codes). For example, “physical health,” “mental health,” and “health” were coded as three separate units. Through this coding process, a total of 750 responses were generated, comprising 327 unique linguistic units, with an average of 6.1 units per participant.

Next, adapting analyses conducted by Hone et al. (2015) and Bharara et al. (2019), we condensed the 327 linguistic units into categories. Linguistic units with different grammatical forms of the same word or that had similar meanings were combined into the same category. For instance, “happy” and “happiness” were combined into the category “happiness.” The linguistic units “comfortable,” “pleasure,” and “relax” were combined into the category “feeling good.” Throughout this process, we aimed to balance maximizing the representation of authenticity of responses while avoiding redundancy. For instance, one participant included the phrase “speaking speed.” We maintained this category to show its distinctiveness as a cognitive indicator. In contrast, we judged other words and phrases to be representative of a single cognitive indicator. For instance, we combined “flowers and plants,” “beach,” and “sunny day” into the category of “nature and beauty.”

Finally, we grouped categories with similar meaning into representative components. For example, the categories “confidence,” “courage,” “curiosity,” “resilience,” “self-compassion,” and “self-knowledge” were grouped into the component “self-strength.” The categories “eat and food,” “exercise,” “fit,” “regular lifestyle,” and “sleep” were combined into the component “physically function well.”

#### Step 2

Step 2 examined the centrality of the wellbeing components identified in Step 1. In contrast to other prototype analyses of wellbeing components, which attempt to identify nomothetic aspects of wellbeing across a population, our analyses represent idiographic notions of centrality (that is, the centrality of components identified by the individual, rather than components identified by others from the population). Our analyses thus accommodated the fact that participants ranked their own components, rather than components identified by others. The following process was used to calculate a score for every component for every participant:

1. Participants rank ordered their own responses, with the option of listing up to 10 linguistic units (which subsequently were converted into components in step 1). We converted ranks to points: points = 11 – rank.
2. When a participant listed multiple components in the same item (e.g., “feeling good, happiness”), that was treated as two separate components with equal rank.
3. When a participant listed the same component twice or more, the higher rank was used.
4. Components listed by a participant were re-ranked, with equal ranks assigned the mean value (this is necessary so that every participant’s points sum to the same value).
5. A participant who listed 10 components would have assigned a total of 55 points (1 + 2 + … + 10 = 55). For each participant, the number of remaining points was calculated as 55 minus the number of points allocated. The remaining points were distributed evenly among all components not listed by that participant.
6. For each component, we calculated the mean and standard deviation of the assigned points.

#### Step 3

Step 3 asked participants to describe a person high in wellbeing and a person low in wellbeing. Narratives were written in English and/or Chinese. To minimize possible distortions in meaning, data were analyzed in the given language, and then results were translated to English, assisted by a NAATI certified translator. The coding and categorizing procedures were similar to Step 1. First, the monolexemic linguistic units in the high level of wellbeing and low level of wellbeing narratives were identified and extracted separately. 392 linguistic units were identified for high wellbeing narratives, with an average of 4.36 linguistic units per participant, and 316 linguistic units were identified for low wellbeing narratives, with an average of 3.51 linguistic units per participant.

As in Step 1, linguistic units were grouped into categories and then components, resulting in 26 components for the high wellbeing narratives and 19 components for the low wellbeing narratives. As in Step 1, coding was conducted by the first author, and then reviewed and finalized with the second and third authors. Finally, Cohen’s kappa was calculated to explore the extent to which high versus low wellbeing descriptions aligned with the conceptualizations of wellbeing identified in Step 1.

## Results

### Step 1: Identifying wellbeing components

The first step asked participants to freely list what they perceived as components of wellbeing. As summarized in Table 2, 30 components were identified. Over half of the participants (61%) considered positive relationships as a component of wellbeing, followed by security (48.0%), then feeling good, physical health, and health (28.5%). Spiritual health, cognitive function, and speaking speed were the least common components, noted by less than 4% of participants.

**TABLE 2:** Wellbeing components, as freely listed by participants.

Component	Frequency (%)	Total participants (%)
Positive relationships	119 (16.7%)	75 (61.0%)
Security	76 (10.6%)	59 (48.0%)
Feeling good	43 (6.0%)	35 (28.5%)
Physical health	39 (5.5%)	35 (28.5%)
Health	34 (4.8%)	35 (28.5%)
Happiness	34 (4.8%)	30 (24.4%)
Achievement and fulfillment	29 (4.1%)	25 (20.3%)
Absence or less negative states	29 (4.1%)	25 (20.3%)
Physically function well	33 (4.6%)	22 (17.9%)
Positivity and optimism	27 (3.8%)	21 (17.1%)
Self-strength	25 (3.5%)	21 (17.1%)
Recreation	25 (3.5%)	19 (15.4%)
Mental health	19 (2.7%)	18 (14.6%)
Motivated and goal driven	22 (3.1%)	16 (13.0%)
Productivity	19 (2.7%)	15 (12.2%)
Calm and peace	17 (2.4%)	15 (12.2%)
Social support network	15 (2.1%)	15 (12.2%)
Satisfaction and contentment	14 (2.0%)	13 (10.6%)
Autonomy and freedom	14 (2.0%)	13 (10.6%)
Good socio-economic environment	13 (1.8%)	11 (8.9%)
Stability	11 (1.5%)	11 (8.9%)
Sense of worth and value	11 (1.5%)	9 (7.3%)
Social cohesion	11 (1.5%)	9 (7.3%)
Meaning and purpose	6 (0.8%)	6 (4.9%)
Energetic	6 (0.8%)	6 (4.9%)
Prosperity	6 (0.8%)	6 (4.9%)
Nature and beauty	5 (0.7%)	5 (4.1%)
Spiritual health	4 (0.6%)	4 (3.3%)
Cognitive function	3 (0.4%)	3 (2.4%)
Speaking speed	1 (0.1%)	1 (0.8%)

### Step 2: Centrality of wellbeing components

**FIGURE 1:**
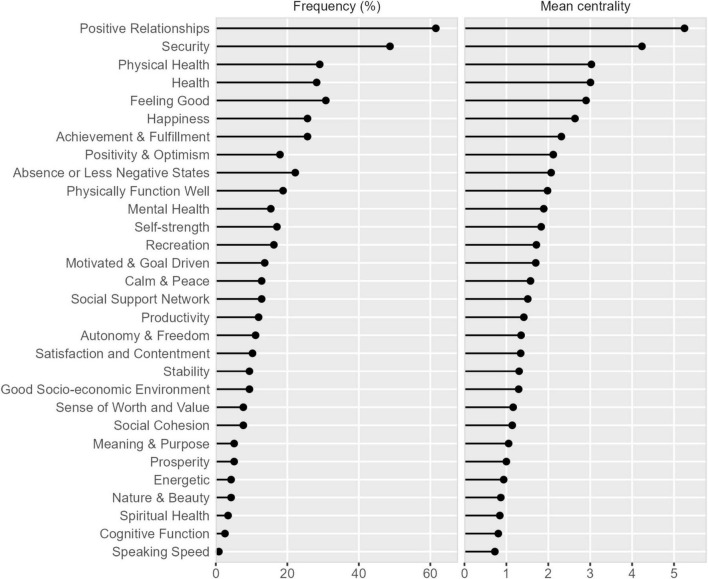
Frequency of component mention versus centrality of components.

Step 2 identified how central the identified components were for the individual respondents. As summarized in Table 3, positive relationships, security, physical health, health, and feeling good were both frequently endorsed and were perceived as central to wellbeing, whereas energetic, nature and beauty, spiritual health, cognitive function, and speaking speed were infrequently mentioned and less central. As illustrated in Figure 1, components that were more frequently mentioned were also more likely to be considered central to wellbeing, whereas less mentioned components were also considered less central to wellbeing. However, there were some exceptions: physical health, health, happiness, positivity/optimism, mental health, and sense of worth/value were less frequently mentioned, but when mentioned were considered more central to wellbeing.

**TABLE 3:** Centrality of wellbeing components, arranged by mean in descending order.

Component	Frequency	Indicators of centrality						
		Mean	SD	Min	Q1	Median	Q3	Max
Positive relationships	72 (62%)	5.2	3.8	0.1	1.0	6.5	9.0	10.0
Security	57 (49%)	4.2	3.9	0.1	0.8	1.6	8.0	10.0
Physical health	34 (29%)	3.0	3.7	0.1	0.6	1.0	7.0	10.0
Health	33 (28%)	3.0	3.8	0.0	0.4	0.8	7.0	10.0
Feeling good	36 (31%)	2.9	3.4	0.0	0.6	1.0	6.0	10.0
Happiness	30 (26%)	2.6	3.4	0.0	0.4	0.8	5.0	10.0
Achievement and fulfillment	30 (26%)	2.3	2.9	0.0	0.4	0.8	3.0	10.0
Positivity and optimism	21 (18%)	2.1	3.2	0.0	0.4	0.8	1.3	10.0
Absence or less negative states	26 (22%)	2.1	2.8	0.0	0.4	0.8	1.3	10.0
Physically function well	22 (19%)	2.0	2.8	0.0	0.4	0.8	1.3	10.0
Mental health	18 (15%)	1.9	2.9	0.1	0.4	0.8	1.3	10.0
Self-strength	20 (17%)	1.8	2.6	0.0	0.4	0.8	1.3	10.0
Recreation	19 (16%)	1.7	2.5	0.0	0.4	0.8	1.3	10.0
Motivated and goal driven	16 (14%)	1.7	2.6	0.0	0.4	0.8	1.3	10.0
Calm and peace	15 (13%)	1.6	2.4	0.0	0.4	0.8	1.0	10.0
Social support network	15 (13%)	1.5	2.3	0.0	0.4	0.8	1.3	10.0
Productivity	14 (12%)	1.4	2.1	0.0	0.4	0.8	1.3	10.0
Autonomy and freedom	13 (11%)	1.4	2.0	0.0	0.4	0.8	1.3	10.0
Satisfaction and Contentment	12 (10%)	1.3	2.0	0.0	0.4	0.8	1.0	9.0
Stability	11 (9%)	1.3	2.0	0.0	0.4	0.8	1.0	10.0
Good socio-economic environment	11 (9%)	1.3	2.0	0.0	0.4	0.8	1.0	10.0
Sense of worth and value	9 (8%)	1.2	1.7	0.0	0.4	0.6	1.0	8.5
Social cohesion	9 (8%)	1.1	1.7	0.0	0.4	0.6	1.0	9.5
Meaning and purpose	6 (5%)	1.1	1.7	0.0	0.4	0.6	1.0	10.0
Prosperity	6 (5%)	1.0	1.5	0.1	0.4	0.6	1.0	8.0
Energetic	5 (4%)	0.9	1.4	0.0	0.4	0.6	1.0	10.0
Nature and beauty	5 (4%)	0.9	1.0	0.0	0.4	0.6	1.0	7.0
Spiritual health	4 (3%)	0.8	1.0	0.0	0.4	0.6	1.0	6.0
Cognitive function	3 (3%)	0.8	1.0	0.0	0.4	0.6	1.0	9.0
Speaking speed	1 (1%)	0.7	0.7	0.0	0.4	0.6	1.0	7.0

Frequency of a component is reported as number (percentage).

### Step 3: Conceptualizations of high versus low wellbeing

Step 3 further explored participants’ conceptualizations of wellbeing, based upon narratives describing high versus low wellbeing. As summarized in Table 4, 26 components emerged from the high wellbeing narratives. Security, positive relationships, self-strength, physical health, and positivity/optimism were the top five components, each mentioned by more than 20% of participants. Energetic, calm and peace, spiritual health, and meaning/purpose were mentioned by less than 5% of participants.

**TABLE 4:** Wellbeing components evident in participants’ descriptions of high wellbeing.

Component	Frequency (%)	Total participants (%)
Security	39 (10.30%)	37 (41.1%)
Positive relationships	58 (15.30%)	36 (40.0%)
Self-strength	26 (6.90%)	21 (23.3%)
Physical health	22 (5.80%)	21 (23.3%)
Positivity and optimism	22 (5.80%)	20 (22.2%)
Absence or less negative states	21 (5.50%)	19 (21.1%)
Happiness	19 (5.00%)	19 (21.1%)
Achievement and fulfilment	17 (4.50%)	15 (16.7%)
Motivated and goal driven	18 (4.70%)	14 (15.6%)
Physically function well	17 (4.50%)	12 (13.3%)
Recreation	18 (4.70%)	11 (12.2%)
Productivity	12 (3.20%)	11 (12.2%)
Stability	11 (2.90%)	11 (12.2%)
Good socio-economic environment	11 (2.90%)	10 (11.1%)
Mental health	10 (2.60%)	10 (11.1%)
Satisfaction and contentment	9 (2.40%)	9 (10.0%)
Social support network	8 (2.10%)	8 (8.9%)
Health	7 (1.80%)	7 (7.8%)
Autonomy and freedom	6 (1.60%)	6 (6.7%)
Feeling good	6 (1.60%)	6 (6.7%)
Social cohesion	6 (1.60%)	6 (6.7%)
Sense of worth and value	5 (1.30%)	5 (5.6%)
Energetic	3 (0.80%)	3 (3.3%)
Calm and peace	3 (0.80%)	3 (3.3%)
Spiritual health	3 (0.80%)	3 (3.3%)
Meaning and purpose	2 (0.50%)	2 (2.2%)

As summarized in Table 5, compared to the positive narratives of a high level of wellbeing person, participants described a low wellbeing person in a negative way. For the low wellbeing narratives, four components were generated by more than 20% of participants: self-characteristics and weakness, negative relationships, negative emotions/states, and worries/pressure. Meaningless/purposeless, positive emotions/states, social division, underachievement/no progress, and low life satisfaction were mentioned by less than 10% participants.

**TABLE 5:** Wellbeing components evident in participants’ descriptions of low wellbeing.

Component	Frequency (%)	Total participants (%)
Self-characteristics and weakness	40 (12.7%)	27 (30.0%)
Negative relationships	32 (10.1%)	27 (30.0%)
Negative emotion and states	31 (9.8%)	23 (25.6%)
Worries and pressure	25 (7.9%)	21 (23.3%)
Socio-economic environment difficulties	19 (6.0%)	17 (18.9%)
Insecurity	18 (5.7%)	17 (18.9%)
Mental illness	18 (5.7%)	15 (16.7%)
Pessimism and hopelessness	14 (4.4%)	13 (14.4%)
Health functioning issue	16 (5.1%)	12 (13.3%)
Unmotivated and no goals	15 (4.7%)	12 (13.3%)
Disengagement	14 (4.4%)	12 (13.3%)
Financial difficulties	12 (3.8%)	12 (13.3%)
Physical ill-health	13 (4.1%)	11 (12.2%)
Unproductive	13 (4.1%)	11 (12.2%)
Meaningless and purposeless	8 (2.5%)	8 (8.9%)
Positive emotions and states	13 (4.1%)	7 (7.8%)
Social division	7 (2.2%)	7 (7.8%)
Underachievement and no progress	5 (1.6%)	5 (5.6%)
Low life satisfaction	3 (0.9%)	3 (3.3%)

**FIGURE 2:**
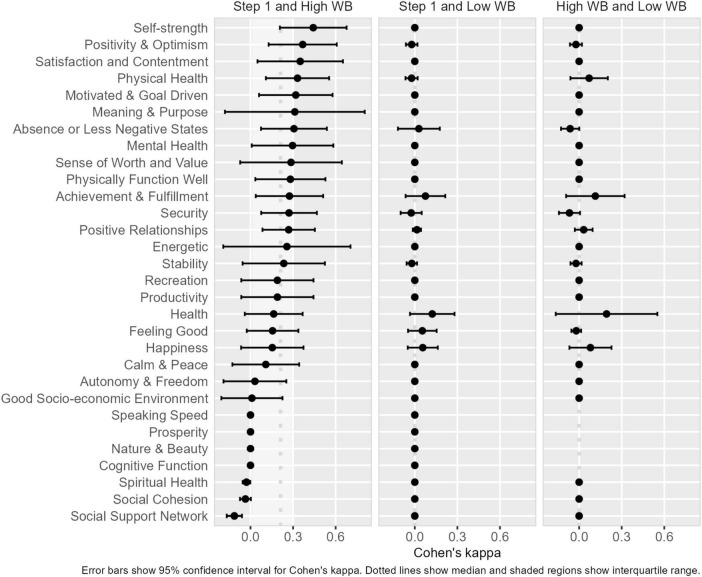
Alignment (indicated by the average Cohen’s kappa and 95% confidence interval) between participants’ conceptualizations of wellbeing indicated in Step 1 with descriptions of high and low wellbeing provided in Step 3. WB = wellbeing.

We next examined the extent to which descriptions of high and low wellbeing from Step 3 aligned with participants’ conceptualizations of wellbeing identified in Step 1. As illustrated in Figure 2 (see Supplementary Material 2 for values), while there was considerable variability across participants, there was some degree of overlap between the Step 1 components and high wellbeing components (median agreement = 0.21, interquartile range = 0.02, 0.29), whereas there was very little overlap between both the Step 1 components and low wellbeing (median agreement = 0.00, interquartile range = 0.00, 0.00), and between descriptions of high and low wellbeing (median agreement = 0.00, interquartile range = 0.00, 0.00).

**FIGURE 3:**
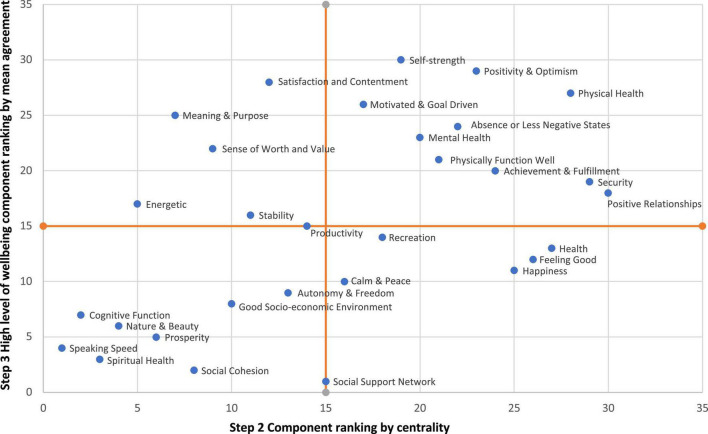
Components’ ranking correlation between the Step 2 component rankings (centrality) and the Step 3 descriptions of a person with high wellbeing (mean agreement).

Finally, we examined alignment between component centrality, as identified in Step 2, with the wellbeing descriptions in Step 3. For example, self-strength was ranked 12th in Step 2 but first in Step 3. The analysis thus considers importance of a wellbeing component, compared to how much a person with high wellbeing is influenced by that components. As illustrated in Figure 3, 10 components had both high centrality and high mean agreement, including positivity/optimism, physical health, security, and positive relationships. In comparison, eight components in the low centrality and low mean agreement category, which indicate less importance and detachment to their understandings and narratives of this concept.

## Discussion

In recent years, student wellbeing has increasingly become an important topic for tertiary institutions, attracting academic and practical interest at both the individual and institutional level. In this study, we conducted a modified prototype analysis to explore conceptualizations of wellbeing for Chinese international students living in Australia. Through a three-step process, we explored individual and collective perceptions of wellbeing. The resulting prototypical structure of wellbeing included a number of components (e.g., satisfaction and contentment, feeling good, physically function well) with both central components (e.g., physical health, positivity/optimism) and peripheral components (e.g., prosperity, cognitive function). The findings provide insights into lay conceptualizations of wellbeing, and potentially can be used to inform best practice approaches for promoting student wellbeing within tertiary educational institutions.

### Chinese international students’ conceptualizations of wellbeing

In Step 1, 30 components of wellbeing were identified, which is comparable to other prototype studies of psychological constructs such as wellbeing (Hone et al., 2015; Jarden et al., 2018; Bharara et al., 2019), love and commitment (Fehr, 1988), and forgiveness (Kearns and Fincham, 2004). Some of the mentioned components aligned with existing psychologically based academic models of subjective wellbeing (Rogers, 1980; Ryff, 1995; Diener, 2000; Seligman, 2018), including positive relationships, feeling good, happiness, achievement/fulfilment, positivity/optimism, self-strength, motivated/goal driven, social support network, autonomy/freedom, and social cohesion. Other components that appear in psychologically based academic models, such as meaning/purpose, sense of worth/value, and satisfaction/contentment were only mentioned by a handful of participants. Notably, several of the most frequently mentioned components, such as security and physical health, are absent from many of the psychological based academic models of subjective wellbeing. Participants also pointed to self-strength and recreation, perceiving characteristics, strengths, and actions as components of wellbeing, components that are missing from academic models.

Similar to a prior study investigating language about wellbeing used by Chinese international students, participants’ included positive relationships, security, physical health, health, and physically functioning well as part of their conceptualization of wellbeing (Huang et al., 2020). Other studies with Chinese participants similarly emphasize the importance of positive relationships and connection for wellbeing (Zhang and Goodson, 2011; Pang, 2018; Yu and Moskal, 2019). Security reflected a desire for financial security and safe/stable living environment. Other studies have pointed to the difficulties and challenges that students face and the desire for the provision of more financial aid (Yan and Berliner, 2011, 2013; Han et al., 2013; Ching et al., 2017).

Interestingly, both physical health and health were commonly mentioned, whereas other studies had less emphasis on the physical health aspects (Li et al., 2014; Martin, 2020). Further, in the current study, only 14.6% of participants listed mental health as a component of wellbeing compared to 67% in Huang et al.’s (2020) study. Notably, participants also mentioned the absence of or fewer negative states as a component of wellbeing, suggesting that wellbeing entails not only the presence of positive aspects, but also the absence or reduction of negative aspects of life.

### Wellbeing as a prototypically organized construct

The Step 2 data demonstrated that some components were considered more central than others, which, according to Rosch (1975) conditions, indicates that participants’ conceptualizations of wellbeing align with a prototypical structure. For example, participants viewed positive relationships, security, physical health, health, and feeling good as more central to wellbeing than speaking speed, cognitive function, spiritual health, nature/beauty, and energy. This aligns with other prototype analyses of laypeople’s’ conceptualizations (Hone et al., 2015; Jarden et al., 2018; Bharara et al., 2019). The results further support that the “fuzzy collection of features” requires considering possession of central components when conducting wellbeing assessment (Lambert et al., 2009, p. 1195).

In general, there was alignment between the frequencies that components were mentioned in Step 1, and the centrality of different concepts identified in Step 2. This aligns with other prototype analyses of wellbeing (Hone et al., 2015; Bharara et al., 2019), suggesting that some components of wellbeing are more universally applicable across a population, whereas other components are more specific to individuals.

### Dual continuum notions of wellbeing

Step 3 pointed to the importance of considering wellbeing from a dual continuum perspective. In the conventional paradigms about wellbeing, the construct exists on a single continuum, and emphasis is placed on promoting positive ends of that continuum (Huppert and So, 2009; Dodge et al., 2012). Alternatively, Keyes and colleagues have emphasized a dual continuum model, in which mental health and mental illness represent two separate dimensions (Westerhof and Keyes, 2010). When participants considered a person with the high level of wellbeing, their narratives aligned with their understanding of wellbeing. However, while some of the low level of wellbeing narratives were described as the opposite of wellbeing (e.g., self-strength/self-weakness, security/insecurity, and mental health/mental illness), other components did not lie on this single continuum. Representing a second continuum, participants mentioned various degrees of emotional and life states in the comparison of high and low level of wellbeing. For example, absence/fewer negative states were highlighted as a key component in the high-level wellbeing narratives while positive emotions/states were mentioned in the narratives of low-level wellbeing. Also, wellbeing conceptualizations included both positive and negative emotional/cognitive functioning. The narratives of high-level wellbeing aligned with the perception of wellbeing and mental health, whereas the narratives of low-level wellbeing were more related to mental illness. This finding suggests that greater attention might be given to a dual nature of wellbeing and mental illness when engaging Chinese students with wellbeing support and service.

### An idiographic approach to prototype analysis

Notably, our study modified other wellbeing-focused prototype analysis studies (Hone et al., 2015; Jarden et al., 2018; Bharara et al., 2019), in that the same participants completed all three steps in a single study, rather than different participants from that population completing the steps across multiple time points, with iterations between the participant responses and processing of that responses by the researchers. While this means that prototypical structure identified may be unique to our sample, rather than representative of Chinese international students living in Australia in general, our modified approach has several specific advantages. Considerable time is saved in the data collection process. It reduces potential historical or time-related factors that might influence responses. There is also less interference by the researcher in the process.

Our modified approach also allows for a more person-centered consideration of wellbeing conceptualizations. Different responses across participants, both in terms of the components identified and the centrality of those components, might indicate diverse cognitive processes (Fehr, 1988), different personal histories and experiences, or other value-based or contextual aspects. This points to the potential value of incorporating not only nomothetic approaches to understanding lay conceptualizations of wellbeing, but also idiographic approaches that capture individual differences within those conceptualizations. Future studies might further examine the advantages and disadvantages of each approach and consider combinations of the two approaches.

### Limitations and future directions

Our study provides some intriguing insights and points to several directions for future research, but also has a number of limitations. As a modified prototype analysis was utilized in this study, the findings could be a result of the method used, rather than a representation of replicable patterns. While a fairly sizeable sample was included, the results might be very specific to this sample. Replication is needed to consider the extent to which results might generalize to other similar samples. As the respondents were allowed to answer the survey in either English or Chinese, it is possible that the translation could be affected by the translator’s personal understanding and perception of the meaning of the Chinese words related to wellbeing. During the translation process, a NAATI register translator assisted to prevent meaning distortion. For the wellbeing words and phrases in Chinese, the data were translated into English first and then did the analysis. For the wellbeing narratives, the checked transcripts were analyzed first and then carefully translated into English. The technique of Double-Translation was also utilized in the translation process to ensure the linguistic equivalence of data in two language versions. The Chinese version of the data was translated into English, then translated back to, and compared with the original Chinese language to identify possible discrepancies. In this way, mistranslations or interpretation errors can be detected and minimized.

This study focused on Chinese international students in the Australian context, which might not generalize to other countries’ context or international students from different cultural backgrounds. Future studies might expand to other populations and contexts to explore similarities and differences amongst international students from different cultures living within different host countries, which can provide guidance for tertiary education institutions in terms of helping international students to thrive. Furthermore, similar to the current study, future studies should examine the impact of the interactions between social contexts, cultural differences, and linguistic backgrounds upon the different lay conceptualizations of wellbeing. For example, refugees might be a population for future exploration. They are not only a minority group relocated to a new social context with different cultural or language backgrounds, but they also have unique experiences of wellbeing, which might lead to different conceptualizations of wellbeing.

The centrality (importance) of the wellbeing components identified potentially might inform guidelines for higher educational institutions on resource allocation in order to offer more efficient and sufficient support for Chinese international students. For example, as participants indicated valuing physical health and security as more central to wellbeing than mental health, tertiary education institutions might pay more attention to providing service related to how to support students maintain a healthy, regular lifestyle, enhance campus security, and offer more work/internship opportunities to complement emphases on psychological counseling services. The components of self-strengths and self-characteristics and weakness mentioned in the high/low wellbeing narratives might point to cultural-based stigmas that align with moralizing dimension of lay understanding, which emphasizes individual responsibility (Haslam, 2005). This might also explain why Chinese students mainly choose intra-personal activities when they intend to maintain or improve their own wellbeing (Huang et al., 2020). Future studies should consider how tertiary educational institutions might best approach students with wellbeing support and services, in ways that minimize stigma and best fit the conceptualizations, values, and needs of students.

## Conclusion

Chinese international students are critical to the Australian economy and culture, and yet are at high risk of experiencing compromised wellbeing and suffering various mental health/illness issues, often not accessing supports that the university may offer. The lay conceptualizations and prototypical structures regarding wellbeing that students have, which are informed by their own cultural background, impact the possible mismatch of service provisions and support structures with students’ wellbeing needs that are less effective and useful as they aim to, especially when those structures misalign with the host culture. To support international students’ wellbeing, it is critical to understand their conceptualizations of wellbeing.

Prototype analysis provides a method to uncover the underlying cognitive structures of wellbeing. Students’ conceptualizations were indeed prototypically organized, suggesting that structured approaches to supported wellbeing can be effective. However, the unique position of students living in a foreign culture and navigating multiple languages helps to broaden Western conceptualizations of wellbeing. The analyses revealed alternate prototypes, which can inform the understanding, assessment, and support of wellbeing across different populations. As a whole, the findings broaden understandings of wellbeing conceptualizations from a lay perspective, provide new data of international students through a wellbeing lens, offer a potential approach to further explore prototype-structured concepts, and provide insights into the nexus of micro and macro aspects that impact upon Chinese international students’ experience of wellbeing.

## Data availability statement

The raw data supporting the conclusions of this article are available on request from the corresponding author.

## Ethics statement

The studies involving human participants were reviewed and approved by The University of Melbourne’s Ethics Committee (protocol #1954456.1). The patients/participants provided their written informed consent to participate in this study.

## Author contributions

LH, MK, and LO: conceptualization. LH and MK: methodology. MK: validation. LH: formal analysis, writing—original draft preparation, and visualization. MK and LO: writing—review and editing and supervision. All authors have read and agreed to the published version of the manuscript.

## Footnotes

1
www.lifeline.org.au
