# Supplementary material for: Chinese international students’ conceptualizations of wellbeing: A prototype analysis
Source: Front Psychol. 2022 Aug 24;13:939576. doi: 10.3389/fpsyg.2022.939576 (PMC9450937; doi:10.3389/fpsyg.2022.939576)
Supplement: Supplementary file 1 [file Data_Sheet_1.PDF]

Supplement 1

Conversions of participants' free response of their English proficiency level

	Comprehensive			Reading			Writing		
**Proficiency Level**	IELTS	TOEFL	PTE	IELTS	TOEFL	PTE	IELTS	TOEFL	PTE
Extremely limited	0-4	0-31	0-29	0–4	0–2	0-29	0–4	0–11	0-29
Limited	4.5	32-34	30-35	4.5	3	30-35	4.5	12–13	30-35
Modest	5	35-45	36-41	5	4–7	36-41	5	14–17	36-41
	5.5	46-59	42-49	5.5	8–12	42-49	5.5	18–20	42-49
Competent	6	60-78	50-57	6	13–18	50-57	6	21–23	50-57
	6.5	79-93	58-64	6.5	19–23	58-64	6.5	24–26	58-64
Good	7	94-101	65-72	7	24–26	65-72	7	27–28	65-72
	7.5	102-109	73-78	7.5	27–28	73-78	7.5	29	73-78
Excellent	8	110-114	79-82	8	29	79-82	8	30	79-82
	8.5	115-117	83-85	8.5	29	83-85	8.5	30	83-85
	9	118-120	86-90	9	30	86-90	9	30	86-90

Note. IELTS = International English Language Testing System, TOEFL = Test Of English as a Foreign Language, PTE = Pearson Test of English

Supplement 2

Cohen's kappa indicating agreement between the wellbeing components identified in Step 1 with descriptions of high and low wellbeing in Step 3, and agreement between high and low wellbeing descriptions.

Component	Step 1-High Wellbeing	Step 1-Low Wellbeing	High Wellbeing-Low Wellbeing
Self-strength	0.44 (0.21, 0.68)	0.00 (0.00, 0.00)	0.00 (-0.00, 0.00)
Positivity & optimism	0.37 (0.13, 0.61)	-0.02 (-0.06, 0.02)	-0.02 (-0.06, 0.02)
Satisfaction and contentment	0.35 (0.05, 0.65)	0.00 (0.00, 0.00)	0.00 (-0.00, 0.00)
Physical health	0.33 (0.11, 0.55)	-0.02 (-0.07, 0.02)	0.07 (-0.06, 0.20)
Motivated & goal driven	0.32 (0.06, 0.58)	0.00 (0.00, 0.00)	0.00 (0.00, 0.00)
Meaning & purpose	0.31 (-0.18, 0.80)	0.00 (-0.00, 0.00)	0.00 (-0.00, 0.00)
Absence or less negative states	0.31 (0.07, 0.54)	0.03 (-0.12, 0.18)	-0.06 (-0.13, 0.00)
Mental health	0.30 (0.01, 0.58)	0.00 (-0.00, 0.00)	0.00 (0.00, 0.00)
Sense of worth and value	0.29 (-0.07, 0.64)	0.00 (-0.00, 0.00)	0.00 (0.00, 0.00)
Physically function well	0.28 (0.03, 0.53)	0.00 (-0.00, 0.00)	0.00 (0.00, 0.00)
Achievement & fulfillment	0.27 (0.04, 0.51)	0.07 (-0.06, 0.21)	0.11 (-0.09, 0.32)
Security	0.27 (0.08, 0.47)	-0.03 (-0.10, 0.05)	-0.07 (-0.14, 0.01)
Positive relationships	0.27 (0.08, 0.45)	0.01 (-0.01, 0.04)	0.03 (-0.03, 0.09)
Energetic	0.26 (-0.19, 0.70)	0.00 (-0.00, 0.00)	0.00 (-0.00, 0.00)
Stability	0.23 (-0.06, 0.52)	-0.02 (-0.06, 0.02)	-0.02 (-0.06, 0.02)
Productivity	0.19 (-0.06, 0.44)	0.00 (-0.00, 0.00)	0.00 (-0.00, 0.00)
Recreation	0.19 (-0.06, 0.44)	0.00 (-0.00, 0.00)	0.00 (-0.00, 0.00)
Health	0.16 (-0.04, 0.37)	0.12 (-0.03, 0.28)	0.19 (-0.16, 0.55)
Feeling good	0.16 (-0.03, 0.34)	0.05 (-0.05, 0.15)	-0.02 (-0.05, 0.01)
Happiness	0.15 (-0.07, 0.37)	0.06 (-0.05, 0.16)	0.08 (-0.07, 0.23)
Calm & peace	0.11 (-0.13, 0.34)	0.00 (0.00, 0.00)	0.00 (-0.00, 0.00)
Autonomy & freedom	0.03 (-0.19, 0.25)	0.00 (-0.00, 0.00)	0.00 (-0.00, 0.00)
Good socio-economic environment	0.01 (-0.21, 0.23)	0.00 (-0.00, 0.00)	0.00 (0.00, 0.00)
Cognitive function	0.00 (-0.00, 0.00)	0.00 (-0.00, 0.00)	—
Nature & beauty	0.00 (-0.00, 0.00)	0.00 (-0.00, 0.00)	—
Prosperity	0.00 (-0.00, 0.00)	0.00 (-0.00, 0.00)	—
Speaking speed	0.00 (0.00, 0.00)	0.00 (0.00, 0.00)	—
Spiritual health	-0.03 (-0.06, -0.00)	0.00 (-0.00, 0.00)	0.00 (-0.00, 0.00)
Social cohesion	-0.04 (-0.07, 0.00)	0.00 (-0.00, 0.00)	0.00 (-0.00, 0.00)
Social support network	-0.11 (-0.17, -0.06)	0.00 (0.00, 0.00)	0.00 (-0.00, 0.00)

Note. Results shown as Cohen's kappa (95% confidence interval). Missing values indicate a component was not mentioned by any participant.
